# Estrogen Protects Vasomotor Functions in Rats During Catecholamine Stress

**DOI:** 10.3389/fcvm.2021.679240

**Published:** 2021-06-16

**Authors:** Lin Zhang, Chenfei Li, Liting Yang, Gabriel Komla Adzika, Jeremiah Ong'achwa Machuki, Mingjin Shi, Qi Sun, Hong Sun

**Affiliations:** ^1^Department of Physiology, Xuzhou Medical University, Xuzhou, China; ^2^Institute of Cardiovascular Disease Research, Xuzhou Medical University, Xuzhou, China

**Keywords:** estrogen, catecholamine stress, vasomotor function, nitric oxide, endothelin-1

## Abstract

The incidence of dysfunctional vasomotor diseases has mostly occurred in postmenopausal women but not in premenopausal women. Hence, this study sought to investigate the impact of estrogen deficiency during catecholamine stress on vasomotor function. Also, attempts were made to utilize estrogen replacement therapy to mitigate the adverse effects (pathological remodeling) of stress on the aortic vessels to preserve vasomotor functions. To do this, female Sprague-Dawley (SD) rats were ovariectomized (OVX) along with sham operations (Sham). Day 14 after OVX operation, 17-estradiol (E_2_) was subcutaneously implanted (OVX+E_2_). Day 35 after operation, stress was induced by isoproterenol (ISO) subcutaneous injections. Clinically relevant blood pressure indexes (systolic, diastolic, and mean atrial blood pressures) were assessed in the rats. Aortic vascular ring tensions were assessed *in vitro* to ascertain the impact of E_2_ on their vasomotor function. Aortic vascular rings (AVRs) from OVX+ISO exhibited a significant increase in contractility in response to phenylephrine than AVRs isolated from Sham+ISO rats. Also, sera levels of nitric oxide (NO) and endothelin-1 (ET-1) and the expression of p-eNOS/eNOS from vascular tissues were ascertained. We demonstrate that, during stress, E_2_ prevented excessive weight gain and OVX rats had higher blood pressures than those in the Sham group. Further, we showed that E_2_ decreases ET-1 expressions during stress while upregulating NO expressions via enhancing eNOS activities to facilitate vasomotor functions. Finally, histological assessment revealed the E_2_ treatments during stress preserved vasomotor functions by preventing excessive intima-media thickening and collagen depositions in the aortic vascular walls.

## Introduction

Premenopausal women have a lower incidence of vascular disease than men of the same age, but this sexual advantage disappears after menopause (1, 2). Stress also tends to cause vascular disease (3, 4). Studies have shown that postmenopausal women have an increased response to stress, and estrogen supplementation can reduce this response (5, 6). This suggests that stress and estrogen deficiency are both risk factors for vascular disease in postmenopausal women.

Research findings over the last decade have demonstrated that by supplementing estrogen during the menopausal period, the adverse effects of catecholamine stress on the cardiovascular system (CVS) are subdued (7, 8). Hence, these findings suggested that experiencing chronic stress during an estrogen deficiency period may be risk factors that facilitate vascular disease pathogenesis in menopausal women. Ensuring good vascular health in the aged is of great public health importance; therefore, considerable efforts have been put into investigating the pathomechanism of these vascular diseases. Regardless, all is still not known.

With the accelerating pace of life, people have to deal with all kinds of pressure, leaving them in a state of stress. In stress state, high levels of circulating catecholamine overstimulate adrenergic receptors, rendering the receptors desensitized or dysfunctional and causes a significant toll on overall health, vascular insults, and impedes proper cardiovascular function (9–11). However, estrogen (E_2_) has been demonstrated to mitigate these adverse effects (12–14).

Studies have shown that hypertension, atherosclerosis, coronary heart disease, and other vascular diseases are closely related to stress, and these diseases are mainly manifested as abnormal vasomotor function (15, 16). The morphological structure of the blood vessels puts the intima (innermost layer of the vessel mainly composed of endothelium) in a position that makes it serve as a permeability barrier in the circulatory system. By this, endothelial cells are able to regulate vasomotor responses of vascular smooth muscle cells by secreting vasoactive substances such as nitric oxide (NO) and endothelin-1 (ET-1). Whereas, the blood vessel media, which is composed of smooth muscle and collagen fibers, are responsible for the relaxation and contraction of blood vessels as well as maintaining normal vascular tension and blood pressure (17). Hence, clinically an increase of intima-media thickness is used in the diagnosis of atherosclerosis disease. This is suggestive that the vascular walls have been stiffened due to the massive deposition of collagen fibers between vascular smooth muscle cells (18). The elasticity of the vascular wall at this point is reduced, and this facilitates the progression and exacerbation of vascular diseases.

Earlier studies have demonstrated that estrogen stimulates endothelial NO synthase (eNOS) and triggers the production and release of NO, an essential vasodilator (19, 20). Also, estrogen has been shown to promote the growth and repair of injured cells (21, 22). Furthermore, it has been suggested that estrogen can inhibit vascular remodeling by subduing maladaptive inflammatory responses (23).

Herein, we sought to elucidate the pathomechanism of vascular diseases in postmenopausal women. We investigated the regulatory roles and mechanism of action of E_2_ on the vasomotor response during catecholamine stress by inducing its deficiency through ovariectomy in rats.

## Materials and Methods

### Animals

Adult female Sprague-Dawley rats, 180–200g, 2.0 to 2.5-month-old, clean grade, were purchased from the experimental animal center of Xuzhou Medical University [China, license number: SYXH (su) 2002–0038]. Rats were group-housed in standard shoebox cages at a temperature of 23 ± 2°C and humidity of 42–55% controlled environment with a 12 h dark-light cycle. Rats were fed on pelleted food and water ad libitum. The experimental design was approved by the animal ethics committee of Xuzhou Medical University.

### Bilateral Ovariectomy and Sham Operations

After anesthetization, rats were fixed in the prone position and caudal half-back disinfected with povidone-iodine. Subcutaneous incision (about 1.0–2.0 cm) was made in the left-back skin (1.5 cm outside the spine and 1.5–2.0 cm above the femoral head) and the peritoneum cut. The left ovary (red cauliflower-like milky white fat) was located into the abdominal cavity, the left ovarian artery was ligated, and the left ovary excised. The fallopian tube and the surrounding fat had antibiotic (penicillin) dressings and were returned into the abdominal cavity. This was followed by peritoneum and skin suturing. The same procedure was repeated for the right ovary. The sham operations had only subcutaneous incision made in their skin and peritoneum (left and right dorsal sides) without ovaries being excised, followed by antibiotic dressings and suturing. The day of surgery was dated day 0 after surgery. Postoperative intraperitoneal injection of penicillin 80,000 IU was performed for the next 3 consecutive days.

### Experimental Groups

Rats were randomly divided into 6 groups: (1) sham operation (Sham) group: laparotomy without ovary resection, (2) bilateral ovariectomy (OVX) group: bilateral ovaries were removed through the abdominal cavity, (3) OVX+E_2_ group: day 14 after OVX operation, estrogen was injected subcutaneously (s.c.) for 31 days, (4) Sham+ISO group: day 35 after the sham operation, ISO was injected s.c. for 10 days, (5) OVX+ISO group: day 35 after OVX operation, ISO was injected s.c. for 10 days and (6) OVX+E_2_+ISO group: day 14 after OVX operation, E_2_ was injected s.c. for 31 days followed by ISO injections s.c. for 10 days, starting from day 35 after OVX operation. The control group had placebo (vehicle) injections s.c., 40μg/kg/day of E_2_ (E2758; Sigma) and 5mg/kg/day of ISO (160504; Sigma) were the dosages used in the animal models.

After the models were finished, the weights of rats were recorded, and thoracotomies were performed to excised the heart to check heart weights.

### Caudal Arterial Pressure

Rats were then transferred to the experimental room half an hour in advance and allowed to adapt to the experimental environment to ensure they were in a relatively calm state during the measurement. The intelligent non-invasive blood pressure (BP) gauge bp-98A (Beijing Ruanlong biotechnology co., LTD) was set up ready for record BP, and the insulation temperature was set to 38°C. Next, the rats were kept calm and still while leaving their tails exposed. The pressurizer was fixed onto the tails, and blood pressures were measured (*n* = 8). Each rat was measured at least thrice during the modeling period.

### Tension of Aortic Ring

Rats were sacrificed, and the thoracic aortas were isolated for vascular reactivity experiment (*n* = 6). Briefly, four adjacent segments of 2 mm long aortic vascular rings (AVRs) were cut from the thoracic aorta and were hanged in two opposite hooks dipped into tissue bath stations. The two opposite metal hooks were attached to silk thread tension transducers, and aortic ring tensions data were acquired with a multi-channel physiological signal acquisition processing system (RM6240; Chengdu instrument). The tissue bath stations contained prewarmed (37°C) 7 ml of phosphate-buffered solution (PSS) (constituent in mM: 118 NaCl, 25 NaHCO_3_, 4.8 KCl, 2.5 CaCl_2_, 1.2 MgSO_4_, 1.2 KH_2_PO_4_ and 11 glucose). The pH was adjusted to 7.4, the buffer was constantly perfused with mixture gas (95% O_2_/5% CO_2_), and the temperature was maintained at 37°C.

The VRs were allowed to adapt for few minutes in the PSS, then a preload of 1.5 g was recorded for 1 h to monitor stability in the experimental set-up. During this period, the PSS every 15 min. VRs were then contracted with 10^−6^mol/L of phenylephrine (PE) (HY-B0471; MCE). This was followed by relaxation of the VR with 10^−6^mol/L of acetylcholine (ACh) (HY-B0282; MCE). If relaxation to ACh was more than 50% of the contraction, the rings were considered endothelium-intact (24). Next, after repeated washings, rings were exposed to PE (10^−9^-10^−4^mol/L), and PE-induced arterial ring shrinkage effect was observed. Finally, when a stable VR tension was attained at the highest dosage of PE (10^−4^mol/L), 10^−9^-10^−4^mol/L of ACh (cumulative administration) was used to assess the vasodilating effect of ACh.

### Determination of Sera Nitric Oxide

NO is active in chemical properties, and it is metabolized into NO^2−^ and NO^3−^ *in vivo*, and NO^2−^ is further converted into NO^3−^. This method uses nitrate reductase to reduce NO^3−^ to NO^2−^ specifically^−^(A013-2; Nanjing Jiancheng Bioengineering Institute). The NO reaction products in sera were quantified by nitrate reductase method according to the manufacturer's specified procedures, and the absorbance of nitrite was determined at 550 nm by enzyme reader (*n* = 6) (25).

### Assessment of Sera Endothelin-1

Sera ET-1 levels were detected by ELISA (CSB-E06979r; Cusabio) (*n* = 6). All reagents were prepared at room temperature (RT) (18–25°C) for 30 min before use. The assays were done strictly according to the manufacturer's protocol. In brief, all reagents, apparatus, and samples were allowed to resume room temperature. The standard reagent was prepared, and both the standards and samples had 200 μl added to their respective wells and incubated at 37°C for 2 h. Next, the contents of the wells were discarded and dried; without washing, 100 μl of Biotin-labeled antibody solution was added. The plate was sealed and incubated at 37°C for 1 h. Afterward, the contents of the wells were discarded and dried, and each well was washed 3x with 250 μl washing buffer. 100 μL of horseradish peroxidase was added to each well, and the plate was again incubated at 37°C for 1 h. Again, the contents of the wells were discarded and dried, and each well was washed 5x with 250 μl washing buffer. Finally, 90 μl of substrate solution was added to each well for 30 min in the dark, followed by adding 50 μl of the termination solution. The optical density (OD) values were obtained at 420 nm wavelength within the next 5 min.

### Western Blot

Rat aortic vascular tissues were homogenized, lysed, and proteins were extracted (*n* = 3). Proteins were run through gel electrophoresis and transferred onto the membrane as earlier described (26). Non-specific antibody binding was blocked with 4% non-fat milk, and the membranes were incubated overnight at 4°C with the following primary antibodies: eNOS (sc-376751; SANTA CRUZA), p-eNOS (^#^9571; Cell Signal technology), and β-actin (TA-09; ZSGB-BIO). Subsequently, the membranes were incubated in Goat anti-Mouse IgG (ZB-2305, ZSGB-BIO) and Goat anti-Rabbit IgG (ZB-2301; ZSGB-BIO) secondary antibodies, respectively, for 2 h at room temperature. The immunoblotted imaging was done using enhanced chemiluminescence (Tanon, Shanghai, China). Protein bands were quantified with ImageJ software (NIH, Bethesda, MD, USA) and normalized with their β-actin expressions.

### Histological Assessment of Blood Vessels and Surrounding Tissues

Excised thoracic aortas (divided into 3 mm long segments) were washed in 4°C prechilled PBS and blotted with filter paper. Next, the aortic specimens were fixed in 4% paraformaldehyde for more than 24 h, embedded in paraffin and were sectioned at 3 μm thickness.

The tissue sectionings were deparaffinized prior to performing hematoxylin and eosin (H&E), Masson trichrome, and immunohistochemical (IHC) staining. H&E staining was done as previously described to assess the intima-media thickness in the blood vessel across all groups. Masson's trichrome staining was performed as previously described to assess the extent of fibrosis in the vascular walls.

Type I and III collagens IHC staining were done as follows; Antigen retrieval was done using citrate butter (heated at 60°C for 30 min). Blocking non-specific antibody binds was done using H2O2 for 10 min, followed by 3% BSA for 30 min. The vascular tissues were then incubated with type I collagen (14695-1-AP; Proteintech) and type III collagen (22734-1-AP; Proteintech) primary antibodies at 4°C overnight. Next, the tissues were incubated in biotin goat anti-rabbit IgG and streptavidin peroxidase (PV-900; ZSGB-BIO) for 1 h at room temperature. This was followed by DAB staining and hematoxylin staining. Lastly, the tissues were dehydrated and mounted for imaging. The stained IHC sections were analyzed with Image-pro Plus 6.0 Image analysis software (Media Cybernetics; CA; United States). Six visual fields of each section were randomly selected to estimate gray values, and the mean value of gray value for each group was used in the graphical plots.

### Statistical Analysis

All data were analyzed with GraphPad Prism (Prism Version 8.0.2; Graph Pad Prism Software Inc., San Diego, USA) and were presented as mean ± SEM. Comparison between multiple groups was performed by one-way ANOVA, and comparison between two groups was performed by *t*-test, with *P* < 0.05 indicating a significant difference.

## Results

### Estrogen Prevents Excessive Body Weight Gain During Catecholamine Stress

**Figure 1:**
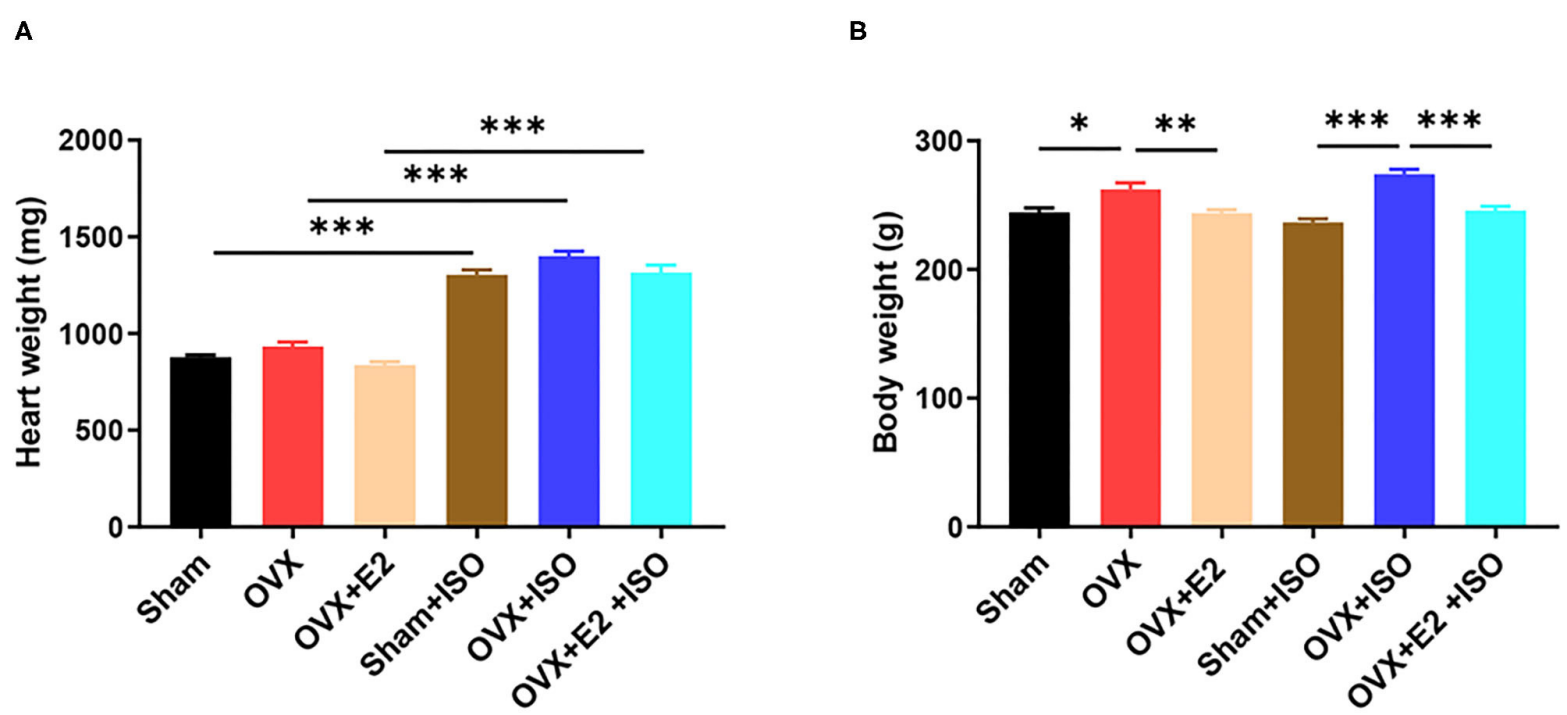
Estrogen prevents excessive body weight gain during catecholamine stress. **(A)** The heart weight. **(B)** The body weight. Results are presented as mean ± SEM. *N* = 8. ^*^P < 0.05, ^**^P < 0.01, and ^***^P < 0.001.

The morphometrics results obtained demonstrate that, in the normal state, the presence of endogenous estrogen (E_2Endo_) in the Sham group and the OVX supplemented with exogenous estrogen (E_2Exo_) in the OVX+E_2_ group influenced reduction in heart weights compared with the OVX group, although there was not any statistical significance among the groups. Also, although the administration of ISO increased the heart weight across all the groups, the effect of E_2Endo_ and E_2Exo_ showed the same trend as under the normal state in comparison with the OVX+ISO (Figure 1A, *P* < 0.001). However, in terms of body weights, it was observed that the deficiency of E_2_ in OVX rats encourages weight gain significantly compared to the Sham and OVX+E_2_. The body weight gain was aggravated significantly by catecholamine stress in the rats deficient in E_2Endo_ and E_2Exo_. Nonetheless, the treatments with E_2Exo_ minimized the excessive body weight gain during catecholamine stress with much significance (Figure 1B, *P* < 0.001).

### Estrogen Decreases Blood Pressure During Catecholamine Stress

**Figure 2:**
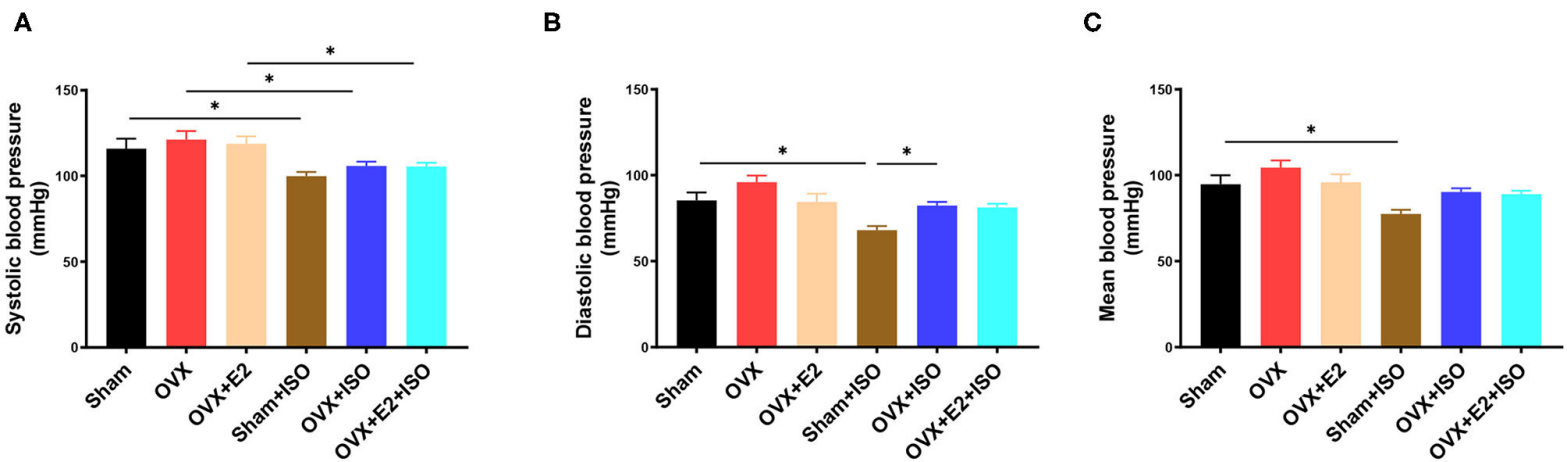
Estrogen decreases blood pressure during catecholamine stress. **(A)** The systolic blood pressure. **(B)** The diastolic blood pressure. **(C)** The mean blood pressure. Results are presented as mean ± SEM. *N* = 8. ^*^P < 0.05.

To explore the effect of estrogen on the blood pressure of rats in stress state, the tail artery pressure was measured *in vivo*. The results showed that in the normal state, the clinically relevant blood pressure indexes, namely; systolic blood pressure, diastolic blood pressure, and mean arterial pressure had no significant differences among the groups, although they were relatively higher in the OVX rats (Figures 2A–C, *P* < 0.05). However, during catecholamine stress, the presence of E_2Endo_ in the Sham+ISO rats decrease the arterial blood pressure indexes more than the E_2Exo_ did in the OVX+E_2_+ISO rats. In the E_2_ deficiency group (OVX+ISO), systolic blood pressure, diastolic blood pressure, and mean arterial remained high (*P* < 0.05).

### Estrogen Decreases Vascular Contractility During Catecholamine Stress

**Figure 3:**
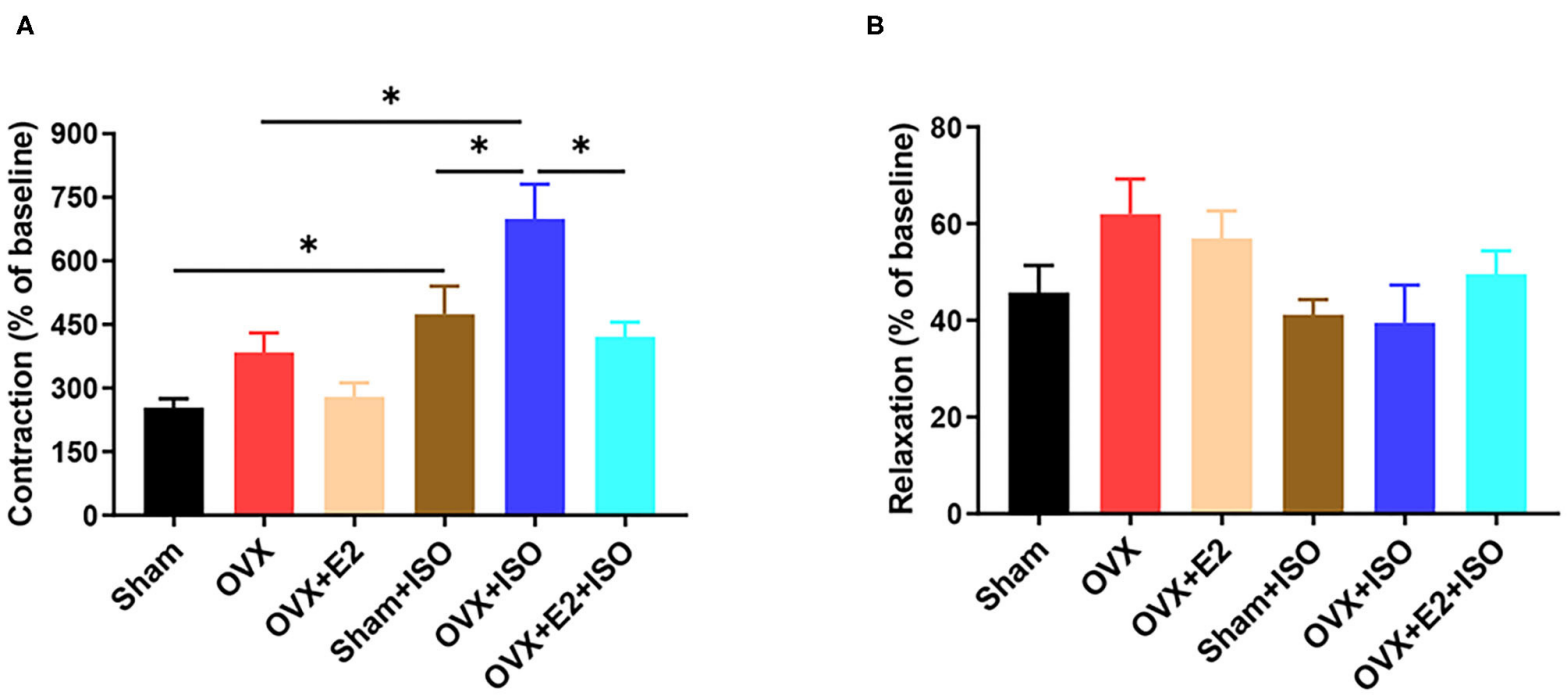
Estrogen decreases vascular contractility during catecholamine stress. **(A)** Effect of estrogen on the contraction in response to PE (10^−4^mol/L) of aortic rings. **(B)** Effect of estrogen on the relaxation in response to ACh (10^−4^mol/L) of aortic rings. Results are presented as mean ± SEM. *N* = 6. ^*^P < 0.05.

*In vivo*, vascular tension is regulated by many factors such as nerves and body fluids. In order to eliminate the interference of nerves and body fluids, we examined the reactivity of rat aortic rings to phenylephrine and acetylcholine *in vitro*. Overall, we observed that the contraction amplitude and the relaxation amplitude of VRs gradually increased in a dose-dependent manner to PE and ACh, respectively. After ISO administration, E_2Endo_ and E_2Exo_ (in Sham+ISO and OVX+E_2_+ISO) supressed the maladaptive contratility observed in OVX+ISO. Also, E_2Endo_ maintained AVRs relaxation better than E_2Exo_ did during stress (Figures 3A,B).

### Estrogen Downregulates Vasoconstrictors and Upregulates Vasodilators

**Figure 4:**
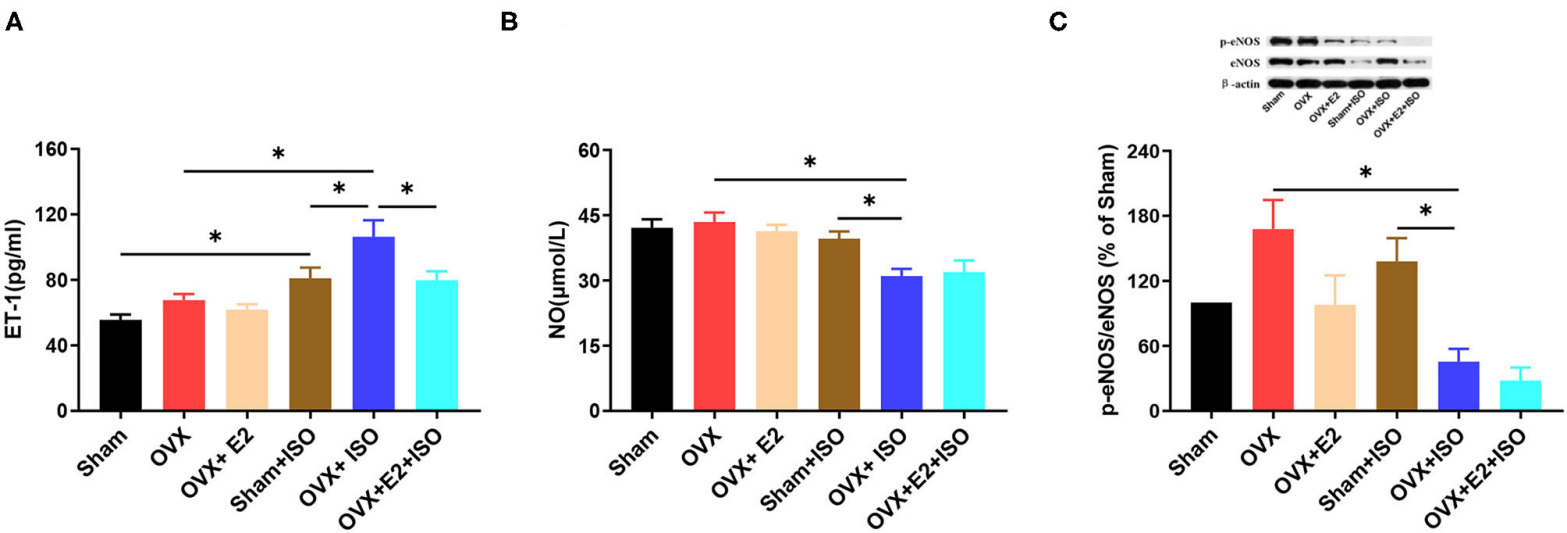
Estrogen downregulates vasoconstrictors and upregulates vasodilators during catecholamine stress. **(A)** The expression of serum ET-1. *n* = 6. **(B)** The expression of serum NO. *n* = 6. **(C)** The protein expression of p-eNOS/eNOS in aortic tissues. *n* = 3. Data are expressed as mean ± SEM. ^*^P < 0.05.

Although under normal state, there were no significant differences observed in sera ET-1 and NO concentrations as well as the protein levels of phosphorylated eNOS (p-eNOS), the results showed a trend. The presence of E_2Endo_ and E_2Exo_ in Sham and OVX+E_2_, respectively, seemed to have decreased the endogenous vasoconstrictor, ET-1. However, during stress, sera ET-1 levels increased significantly in the OVX+ISO group, in comparison with the Sham+ISO and OVX+E_2_+ISO groups (Figure 4A, *P* < 0.05). During stress, the NO (endogenous vasodilator) levels in OVX+ISO and OVX+E_2_+ISO decreased significantly compared to the Sham+ISO (Figure 4B, *P* < 0.05). Protein analysis of p-eNOS in vascular tissues also shown that, during stress, eNOS activities were downregulated significantly in the OVX+ISO and OVX+E_2_+ISO groups, compared to the Sham+ISO group that has E_2Endo_ present (Figure 4C, *P* < 0.05).

### Estrogen Attenuates Intima-Media Thickening During Catecholamine Stress

**Figure 5:**
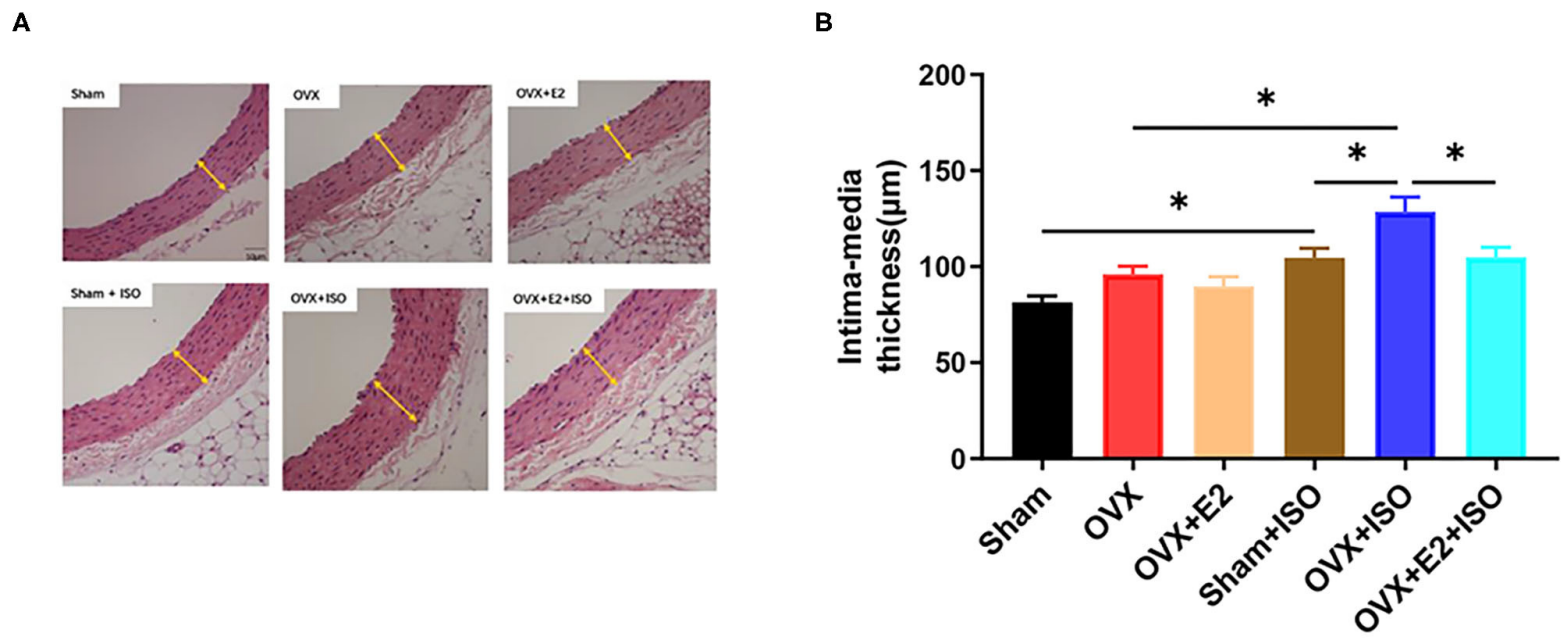
Estrogen attenuates intima-media thickening during catecholamine stress. **(A)** HE staining of tissue sections. **(B)** Quantification of imaging results. Data are expressed as mean ± SEM. *n* = 6. ^*^P < 0.05.Scale bar = 50 μm.

The previous results have suggested that stress has adverse effects on the vasomotor function of OVX rats. To further explore the effect of estrogen on the vascular structure of rats in stress state, rat aorta specimens were sectioned, and HE staining was performed. Examination of stained sections showed that in the normal state, there were no significant differences in aortic intima-media thickness among the groups. Furthermore, during catecholamine stress, aortic intima-media thickness was observed to have increased significantly across all groups (Figures 5A,B, *P* < 0.05). Nonetheless, the presence of E_2Endo_ and E_2Exo_ in Sham and OVX+E_2_, respectively, prevented intima-media thickening during stress significantly on comparing them with the intima-media from OVX+ISO rats (Figures 5A,B, *P* < 0.05).

### Estrogen Decreases Collagen Deposition in Aortic Vascular Walls During Catecholamine Stress

**Figure 6:**
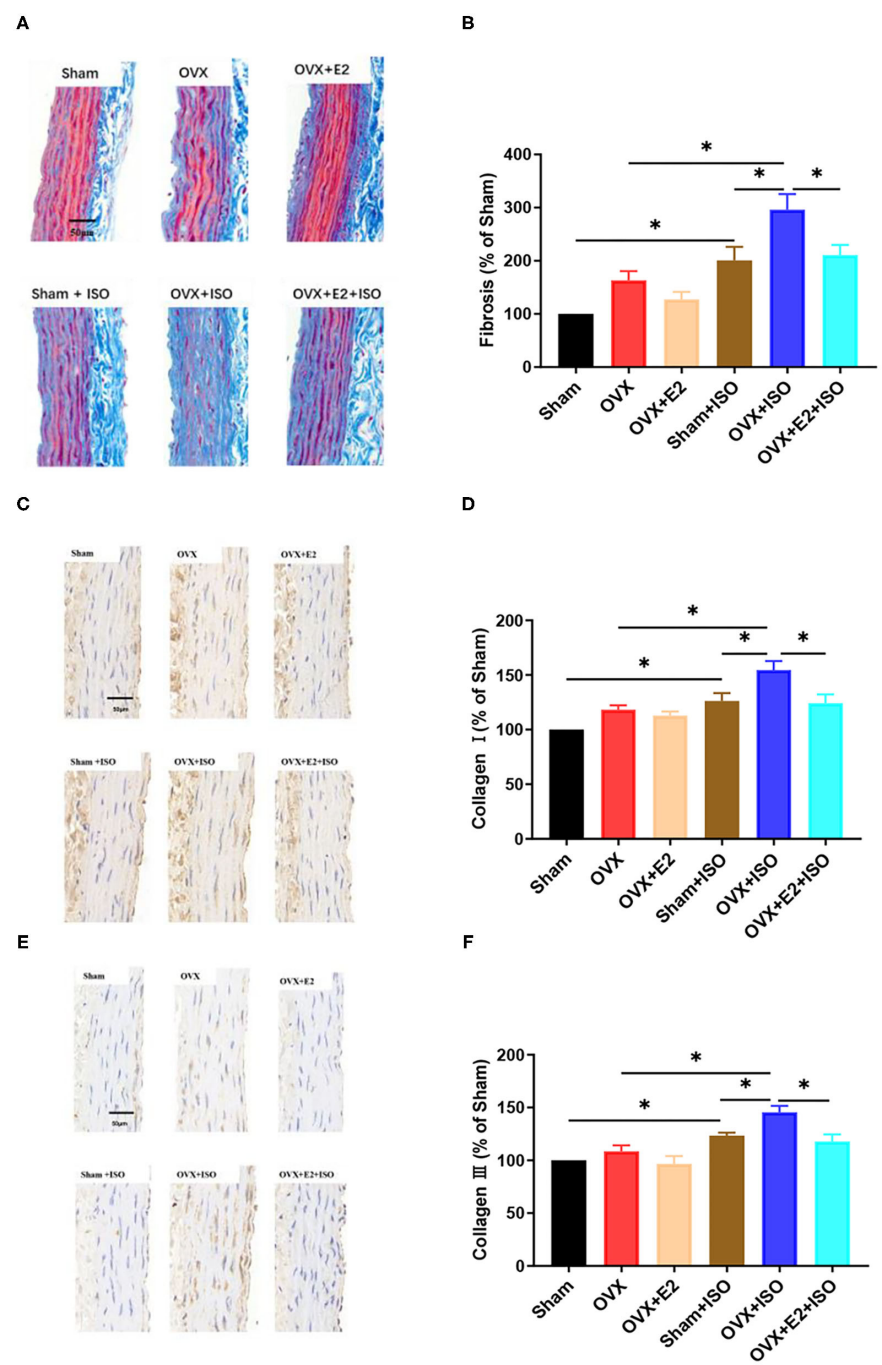
Estrogen decreases interstitial collagen deposition in aortic vascular walls during catecholamine stress. **(A, B)** Representative trichrome staining of collagen depositions in aortic tissues and their quantifications. **(C–F)** Representative IHC staining of type I and III collagens in aortic tissues and their quantifications. Data are expressed as mean ± SEM. *n* = 6. ^*^P < 0.05. Scale bar = 50 μm.

The extent of collagen depositions in aortic vascular walls were assessed across all group with Masson's trichrome staining (Figures 6A,B). The results showed that there were no significant differences in collagen deposition among the groups under normal state. However, during stress, collagen depositions in aortic vascular walls were observed to have increased significantly across all the groups; but the presence of E_2Endo_ and E_2Exo_ in Sham+ISO and OVX+E_2_+ISO, respectively, minimized the extent of the depositions in these groups (Figures 6A,B, *P* < 0.05).

Type I and III collagens are the main collagens expressed in the vascular system; hence, further investigations were done with IHC staining to ascertain if E_2_ has varying effects on their expressions (Figures 6C–F). The IHC staining of the aortic vascular tissues revealed that, under normal state, type I and III collagens expressions had no significant differences among the Sham, OVX, and OVX+E_2_ groups. However, during catecholamine stress, the absence of E_2_ in the OVX+ISO rats significantly increased the expression of type I and III collagens in their aortic vascular walls. Intriguingly, compared to the OVX+ISO rats, an increase in type I and III collagens expressions were significantly minimized by the presence of E_2Endo_ and E_2Exo_ in Sham+ISO and OVX+E_2_+ISO, respectively, during stress (Figures 6C–F, *P* < 0.05).

## Discussion

Clinical demographics demonstrate a higher incidence rate of cardiovascular diseases (CVDs) in men than in women, and this has been widely reported to be due to the protective roles of E_2_ in premenopausal women (27, 28). However, with the decrease of E_2_ level during menopause, women become prone to CVDs just like men of all age cohorts. Furthermore, our modern-day life and its demands elicit catecholamine stress responses that induce adverse cardiovascular remodeling if it continues without any timely intervention (3, 4, 9–11, 29).

Under the physiological state, circulating catecholamine regulate cardiovascular function and homeostasis by activating adrenergic receptors (ARs). Among the subtypes of ARs, β_2_ARs and α1ARs are mainly expressed in the blood vessels; hence, their respective stimulations by catecholamine induce vasodilation and vasoconstriction. This ensures vascular homeostatic function to maintain a healthy tension in the vessels and blood circulation pressure. However, during chronic catecholamine stress (CCS), hyperstimulation of β_2_ARs desensitizes and dysregulates the receptor, causing it to become dysfunctional in mediating stimuli signals to facilitate vasodilation (10). Therefore, during CCS, the vascular homeostatic function is disrupted and builds up the arterial pressure, thereby causing hypertension. Alternatively, E_2_ utilizes multiple mechanisms such as upregulating NO via facilitating eNOS activities and signaling via Angiotensin II receptor type 2 and Mas receptor to ensure vasodilation (20, 30, 31).

Nonetheless, the synergy of hyperstimulation of β_2_ARs and E_2_ deficiency during menopausal aggravates the disruption of the vasomotor, making postmenopausal women vulnerable to hypertension and other cardiovascular complications. Herein, we attempt to elucidate the mechanistic roles of E_2_ in preserving the vasomotor function during catecholamine stress.

By assessing morphometrics at the end of the models, it was observed that the deficiency of E_2_ permitted the excessive gain of body weight resulting from adipose deposition in the rats. This finding adds to the mounting evidence suggesting that E_2_ is essential for effective lipid metabolism. These studies showed that by modulating the activities and expression of low-density lipoprotein receptors (LDLR), scavenger receptor class B type 1 (SR-B1), and proprotein convertase subtilisin/kexin type 9 (PCSK9), E_2_ helps to maintain a healthy balance in the lipid profile by keeping circulating levels of high-density lipoprotein (HDL) high and low-density lipoprotein receptors (LDL) low (20, 32, 33) However, the absence of E_2_ permits a disorder in lipid metabolism, and hence, LDL level increases excessively (34). This results in the deposition of the bad lipid (LDL) in and around organs and vascular tissues in the form of adipose, thereby causing bodyweight gains. The phenomenon above is depicted and consistent with our findings in this study (Figure 1B). OVX rats gained body weight compared to the Sham and OVX+E_2_ rats; however, during stress, the weight gained by OVX+ISO rats increased significantly in comparison with Sham+ISO and OVX+E_2_+ISO. Also, OVX rats' hearts are observed to have increased similarly, probably to help compensate for cardiac function. Besides weight gain, lipid accumulation in the arteries' intimal layer initiates obstructive atherosclerosis, which progresses into coronary artery disease and coronary heart disease. As such, by the possible prevention of lipid depositions in and around vascular tissues, E_2_ might be preserving vasomotor function and minimizing the incidence of atherosclerosis and coronary artery disease in premenopausal women.

We proceeded to assess the impact of E_2_ deficiency on blood pressure. Generally, clinical case reports have demonstrated that premenopausal women's blood pressures are lower than that of men of the same age; however, during menopause, the blood pressure of women increases significantly, which increases the incidence of hypertension (35). Hence, besides aging, E_2_ deficiency during stress has been suggested as a risk factor for women (20). Therefore, we sort to confirm this in our animal models, and the results obtained were consistent with earlier findings (35). It was observed that, although without statistical significance, OVX rats had the highest systolic blood pressure, diastolic blood pressure, and mean arterial, relatively, under normal state. Nonetheless, during ISO-induced stress in the Sham, OVX, and OVX+E_2_, these relevant clinical blood pressure indexes remained relatively highest in the OVX+ISO rats, while the presence of E_2Endo_ (in Sham+ISO rats) significantly decreased them compared to E_2Exo_ (in OVX+E_2_+ISO). As such, adding to the mounting of evidence (36), we demonstrated that the presence of physiological levels of E_2_ decreases blood pressure during catecholamine stress (Figures 2A–C). This might explain why premenopausal women have a lower incidence of developing hypertension while men of their same age cohort and postmenopausal women are predisposed to its occurrence during stress.

Due to other neurohumoral factors, cytokines and enzymes influencing vasomotor functions *in vivo*, aortic vascular ring tension experiments were performed *in vitro* to ascertain the direct impact of E_2_ on the systolic and diastolic responses on the vessels using PE and ACh. Signaling via α-AR, PE is widely used to detect the contractility of AVRs. The results from these experiments showed a significant contractility response of AVRs from OVX+ISO rats to PE, compared to those Sham+ISO and OVX+E_2_+ISO (Figure 3A). The observed hyper-contractility of AVRs from OVX+ISO rats could indicate an ISO-induced vascular endothelial injury and vasomotor dysfunction, as suggested by other studies (37). Similarly, E_2Endo_ (in Sham+ISO) maintained normal relaxations of the AVRs during stress; however, E_2Exo_ was unable to attain this effectively (Figure 3B). Notably, ovarian secretion of progesterone compliments the efforts of E_2_ in ameliorating the adverse effects of stress on the vascular endothelial (38, 39); hence, its absence in OVX+E_2_+ISO may have dampened the efficacy of E_2Exo_ in achieving the aforementioned.

To elucidate the mechanism employed by E_2_ to regulate the blood pressure and mitigate the ISO-induced vascular endothelial injury, we assessed its effect on ET-1 (a prototypical vasoconstrictor) and NO (a prototypical vasodilator). This was done by assessing sera concentration of ET-1 and NO. Besides all the known vasoconstrictors (ET-1, angiotensin II and thromboxane A_2_) secreted by the vascular endothelial cells, ET-1 is the main. Unfortunately, ET-1 is implicated in metabolic disorders and smooth muscle cell proliferation, and its upregulation is deemed a risk factor in vascular injuries and related diseases (40). Hence, under physiological conditions, ET-1 levels are kept in a homeostatic balance with vasodilators such as NO to regulate vasomotor responses (vasoconstrictions and vasodilation) to maintain a healthy blood pressure (41). On assessing sera levels of ET-1, we found that E_2_ deficiency permitted the upregulation of ET-1 highest in OVX rats than in Sham and OVX+E_2_ rats (Figure 4A). This phenomenon was aggravated by the synergy of E_2_ deficiency and stress as ET-1 was shown to have upregulated significantly in the OVX+ISO rats. However, the presence of E_2_ in the Sham+ISO and OVX+E_2_+ISO rats minimized the upregulation of ET-1 (Figure 4A). Also, although vascular endothelial cells secrete prostacyclin and endothelial hyperpolarizing factor besides NO, NO is the essential vasodilator. It performs other functions such as prevention of cell adhesion, platelet aggregation, formation of thrombosis, oxidation of lipoprotein, and smooth muscle cell proliferation (42, 43). These functions impede the initiation and progression of atherosclerotic changes and also facilitates proper vasomotor function (42, 43). However, the deficiency of NO abolishes its vasodilatory, anti-atherosclerotic, and anti-inflammatory effects on the vascular endothelial and vessels, thereby permitting vascular endothelial dysfunction and vascular remodeling during stress, as reported (42–44).

Intriguingly, E_2_ has been shown to stimulate eNOS by inducing its phosphorylation at the ser1,177 site to upregulate NO production and ensure proper vasomotor function (45). Hence, the need to assess this estrogenic effect on NO and eNOS in this stress model. The NO results demonstrated that during stress, E_2_ deficiency in the OVX+ISO rats encouraged a significant decrease in NO levels. Meanwhile, the supplemented E_2Exo_ in the OVX+E_2_+ISO rats was unable to maintain the NO levels as E_2Endo_ did (Figure 4B). Further investigations for p-eNOS protein level revealed that during stress, the E_2Endo_ (in Sham+ISO rats) was able to keep p-eNOS levels up than the E_2_ deficiency (in the OVX+ISO rats) and E_2Exo_ (in the OVX+E_2_+ISO rats) (Figure 4C); hence, explains the observed trend in NO levels among the groups (Figure 4B). In summary, these results showed that E_2_ decreased the blood pressure and mitigated the ISO-induced vascular endothelial injury in the rats during catecholamine stress by enhancing vasodilation via NO upregulation while reduced excessive vasoconstriction via ET-1 downregulation.

Furthermore, histological analysis using HE to assess the vascular morphological changes induced by the synergy of E_2_ deficiency and catecholamine stress revealed that E_2_ is crucial in preventing excessive thickening of the intima-media during stress. In detail, under normal state, the thickness of intima-media of aortic vessels from OVX rats were relatively higher than those from the Sham and OVX+E_2_. During catecholamine stress, rats deficient in E_2_ (OVX+ISO) had their intima-media increased significantly compared to the groups with E_2_ (Sham+ISO and OVX+E_2_+ISO). As clinically suggested, increased intima-media thickness is a manifestation of early atherosclerosis, and the higher the thickness, the higher the incidence of cardiovascular and cerebrovascular diseases (46). Going back to our results, which are in line with the findings of others (47), we hypothesized that the significant intima-media thickening in the OVX+ISO rats might be due to excessive collagen depositions, besides lipid depositions in the vascular walls.

To validate this hypothesis, trichrome staining for fibrosis and IHC staining for type I and III collagens were performed on sectioned aortic vascular tissues. Comparatively, the results showed that E_2_ deficiency enhances fibrosis. This became aggravated during stress, as it is evident by the marked fibrosis (Figures 6A,B); however, E_2_ presence and supplementation in Sham+ISO and OVX+E_2_+ISO, respectively, minimized the extent of the fibrosis in these groups. In addition, type I and III collagens were seen significantly upregulated in the vascular tissues during stress only when E_2_ was deficient in the rats (Figures 6C–F). Massive deposition of type I and III collagens induced by OVX and sustained ISO stimulation have been reported in the myocardium but rarely reported in the vessel (48, 49). Our findings therefore demonstrate that the synergy of stress and E_2_ deficiency aggravates massive collagen deposition in the aortic vessels, which causes vascular stiffness and vasomotor dysfunctions. Nevertheless, E_2_ can mitigate this adverse effect, maintain the elasticity of the vascular wall and delay the onset of vasomotor dysfunctions.

**Figure 7:**
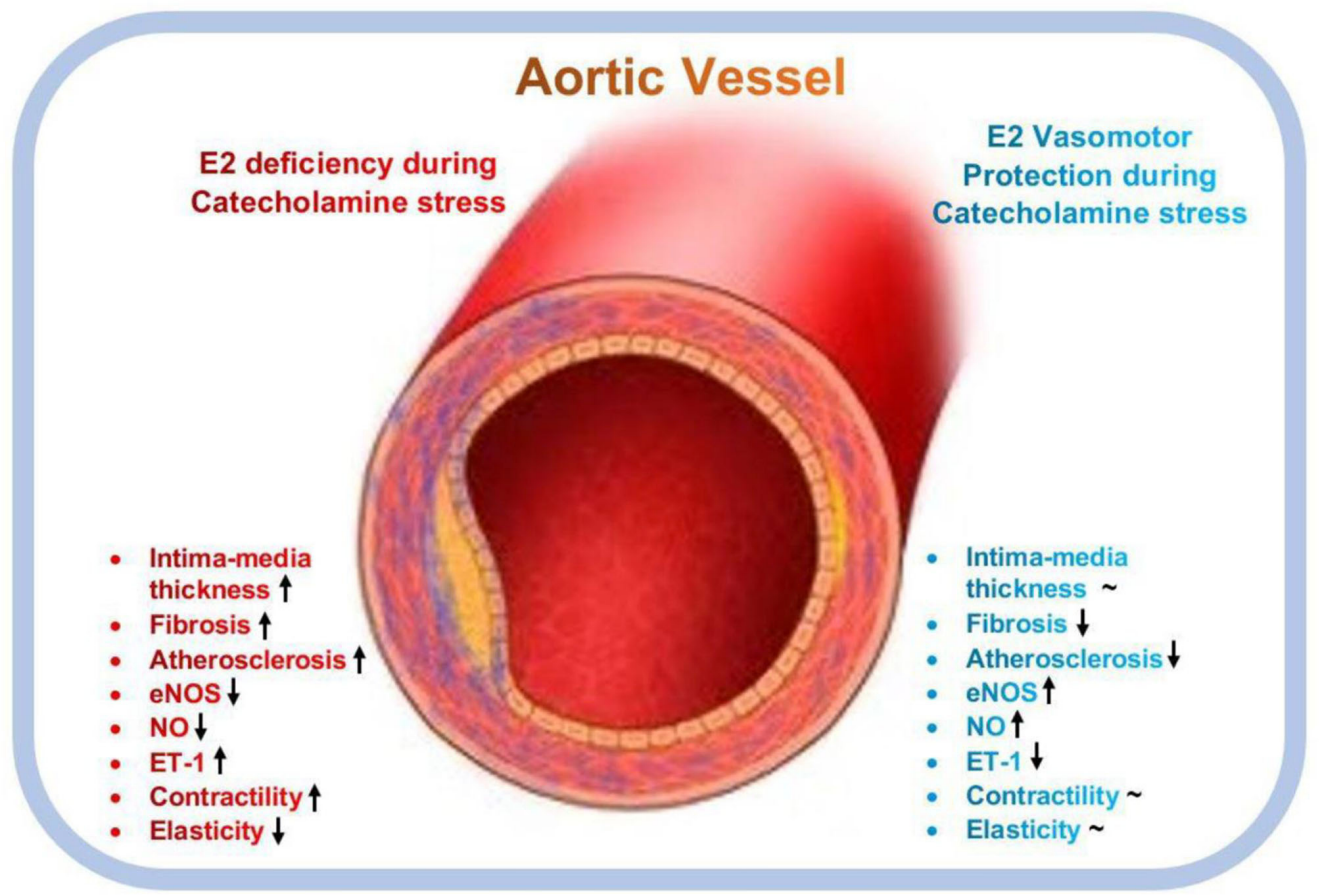
Graphical abstract of the vasomotor function protective roles mediated by estrogen during catecholamine stress. E_2_, Estrogen; eNOS, endothelial nitric oxide synthase; ET-1, endothelial-1; NO, nitric oxide; ↑, upregulates; ↓, downregulates; **~**, normalizes.

In conclusion, this study demonstrates that E_2_ helps to maintain vasomotor function during stress by using multiple mechanisms. E_2_ prevented excessive weight gain in rats, possibly by preventing lipid depositions in and around organs and vessels, hence, impeding the onset of atherosclerosis as suggested (50). Also, E_2_ prevented ISO-induced vascular endothelial injury, adverse vascular remodeling (excessive thickening of intima-media and interstitial collagen deposition in vascular walls), and hypertension in the rats by decreasing blood pressure and vascular contractility during stress via the homeostatic modulation of the expressions and activities of NO and ET-1(Figure 7). These findings reemphasize the need for E_2_ replacement therapy (E_2_RT) in postmenopausal women to minimize the incidence of catecholamine stress-induced vascular disease (51, 52). However, to circumvent the adverse effects of E_2_RT reported by the Women's Health Initiative in their randomized controlled trial (53), it is advised that the administration of menopausal hormone therapy (MHT) to improve cardiovascular health are initiated early within the critical window (5–6 year after menopause) (20, 54). Owing to the clinical significance of this study, it is deemed appropriated to highlight its limitations. In some instances (Figures 2B, 4B,C), it was observed that E_2Exo_ was unable to confer its protective effects as much as E_2Endo_ did. This could be due to the fact that ovaries secrete other hormones and substances to compliment the protective effects of E_2Endo_ in the sham; however, these factors are obliterated by OVX operations. Also, unlike E_2Exo_ dosages, which remained constant throughout the modeling period, E_2Endo_ levels in the Shams do increase and decrease during the estrous cycle (55); hence, E_2_RT must be administered at dosages the mimic its levels in the estrous cycles so as to benefit its vasomotor protective effects during catecholamine stress, fully.

## Data Availability Statement

The original contributions presented in the study are included in the article/supplementary material, further inquiries can be directed to the corresponding authors.

## Ethics Statement

The animal study was reviewed and approved by Laboratory Animal Ethics Committee of Xuzhou Medical University.

## Author Contributions

HS developed the original idea and acted as guarantors. LZ and CL performed animal studies and performed the tension experiments. LZ, LY, and QS conducted the morphology experiments. LZ and MS performed the western blot assay. LZ, GK, and JO wrote the manuscript. All authors contributed to the article and approved the submitted version.

## Conflict of Interest

The authors declare that the research was conducted in the absence of any commercial or financial relationships that could be construed as a potential conflict of interest.
